# Minimization of apoptosis-like changes in cryopreserved buffalo bull sperm by supplementing extender with Bcl-2 protein

**DOI:** 10.14202/vetworld.2016.432-436

**Published:** 2016-05-02

**Authors:** Jasmer Dalal, Ajeet Kumar, Mrigank Honparkhe, Dipak Deka, Narinder Singh

**Affiliations:** 1 Department of Veterinary Gynaecology and Obstetrics, Guru Angad Dev Veterinary and Animal Sciences University, Ludhiana, Punjab, India; 2 School of Animal Biotechnology, Guru Angad Dev Veterinary and Animal Sciences University, Ludhiana, Punjab, India

**Keywords:** anti-apoptotic effects, Bcl-2 protein, buffalo bull, semen cryopreservation

## Abstract

**Aim:**

This study was aimed at evaluating the anti-apoptotic effects of Bcl-2 protein in cryopreserved buffalo bull sperm.

**Materials and Methods:**

A total 10 ejaculates from two buffalo bulls (5 each) were collected using artificial vagina method, and semen was evaluated using a standard protocol. Semen was extended by Tris egg yolk extender supplemented with Bcl-2 protein at 5, 10, and 15 µM. Semen was cryopreserved at ultra-low temperature using traditional vapor freezing method. Pre-freeze and post-thaw semen samples were evaluated for percent motility, viability, hypo-osmotic swelling test (HOST) reactive sperms; status of mitochondrial membrane activity and status of sperm phospholipase A1 and phospholipase A2 activity.

**Results:**

There were no significant effects of Bcl-2 protein supplementation on pre-freeze sperm quality. Percent motility and active mitochondria in post-thaw Bcl-2 supplemented and control groups were also similar. However, viable sperms were significantly (p<0.05) higher (74.29±4.23%) in Bcl-2 supplemented group (5 µM) as compared to control (51.6±5.77%). The proportion of HOST reactive sperms was also higher (63.1±6.73%) in Bcl-2 supplemented (5 µM) group as compared to control (50.7±6.98%). The sperm with low PLA activity (non-apoptotic) was significantly (p<0.05) higher in all the supplemented doses of Bcl-2 protein, i.e., at 5 µM (73.42±5.79%), 10 µM (75.51±6.22%), and 15 µM (74.78±5.89%) as compared to control (60.23±4.45%). We found that Bcl-2 protein supplementation at 5 µM dose improved the post-thaw semen quality indicated by higher viability, HOST reactive sperms, and sperm with low PLA activity (non-apoptotic sperms).

**Conclusion:**

Bcl-2 protein supplementation exerts its protective effect on spermatozoa against apoptosis-like changes developed during cryopreservation.

## Introduction

High quality cryopreserved semen is the primary requirement of dairy sector to achieve high conception rates [1]. One of the factors which decrease the frozen semen quality is the development of apoptosis-like changes in sperm during cryopreservation. Various factors have been attributed to the development of apoptosis-like changes such as cryopreservation, heat exposure, radiation, hydrogen peroxide, genetic disturbances, and endocrine disruptions [2,3]. It has been reported that cryofreezing stress is a major contributing factor to induce apoptosis in sperms by increasing (4.5-54.4%) the proportion of sperm with low mitochondrial membrane potential [4]. Apart from that, several genes and molecules are also responsible for the initiation of apoptosis in sperm such as Bax, Bak, and p53 proteins [5].

It has been established that Bcl-2 family proteins are located in the outer mitochondrial membrane, wherein Bax induces and Bcl-2 inhibits the release of pro-apoptotic proteins, i.e., cytochrome C [6,7]. The release of cytochrome C from the mitochondria leads to DNA fragmentation [4], which is an early marker of apoptosis [8]. It is well established that sperm DNA damage is negatively associated with fertilization rate, implantation, and successful pregnancy [9] and higher occurrences of miscarriage [10]. Further, apoptotic sperms with fragmented DNA and asymmetrical membrane result in poor fertility [11]. It has been well established that apoptosis is executed via various proteins, which are regulated by Bcl-2 family members [8]. Bcl-2 is the most important anti-apoptotic protein and its function is related to interfere with mitochondrial apoptosis pathways [12]. So, the supplementation of anti-apoptotic Bcl-2 protein can inhibit the apoptotic pathways [8,13].

The significance of this study is that the supplementation of Bcl-2 protein might reduce the apoptosis-like changes inflicted during cryopreservation. So, the post-thaw semen quality may be enhanced by minimizing apoptosis of sperms during cryopreservation by supplementation of Bcl-2 protein.

This study was aimed at accessing the ameliorative effects of Bcl-2 protein on apoptosis-like changes in buffalo bull sperm developed during cryofreezing.

## Materials and Methods

### Ethical approval

The approval from the Institutional Animal Ethics Committee to carry out this study was not required as no invasive technique was used. Semen was being collected and frozen as a routine procedure under progeny testing program.

### Selection of buffalo bulls

Two breeding buffalo bull around 4 years of age maintained at the bull farm, Guru Angad Dev Veterinary and Animal Sciences University, Punjab, India (Latitude/Longitude, 30.55°N, 75.54°E) was included for this study. These bulls were under progeny testing program and were being used for semen collection by artificial vagina method. Bulls were maintained under loose housing system (covered area - 12×10 ft and uncovered area - 25×10 ft) and standard feeding schedule along with adlib green fodder.

### Experimental design

Five ejaculates from each buffalo bulls were used in this study. Each ejaculate was extended with Tris egg yolk extender as follows. The anti-apoptotic protein Bcl-2 (cat# Pro-630, Prospec protein specialist) was dissolved in dimethylsulfoxide (DMSO) at 100 μΜ concentration (stock solution). From each extended ejaculates, 4 aliquots were taken. Three aliquots were supplemented with Bcl-2 protein stock solution to make final concentration at 5, 10, and 15 µM. The unsupplemented aliquot was served as control. Semen samples were frozen using traditional vapor freezing method. The quality of pre-freeze and post-thaw semen in terms of % individual motility, % viability, % hypo-osmotic swelling test (HOST) reactive sperms, % active mitochondria, and % sperm with low phospholipase A (PLA) activity (non-apoptotic sperms) was evaluated. Before recording the observations, 5 dummy trials were conducted to standardize the protocol. The incubation time for fluorescent imaging was also standardized accordingly. Thereafter, actual observations were recorded from a total of 10 ejaculates. Moreover, our aim was not to study the apoptosis-like changes due to bull variations.

The % individual motility was assessed manually under 20× objective of phase contrast microscope (Nikon Eclipse E 200). The live sperm count was determined through Eosin-Nigrosin staining technique [14]. The HOST was performed to assess the functional integrity of sperm membrane [15].

### Evaluation of mitochondrial membrane activity in Bcl-2 supplemented pre-freeze and post-thaw semen

**Figure-1:**
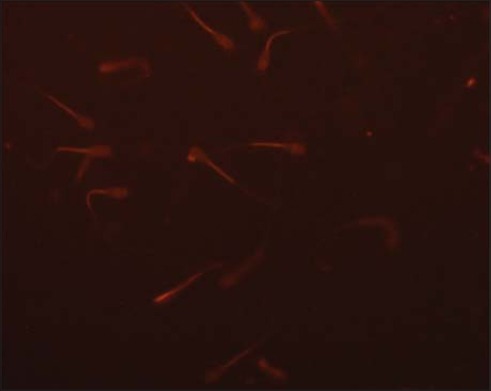
Sperm mitochondria stained with TMRM, TRITC filter (400x).

**Figure-2:**
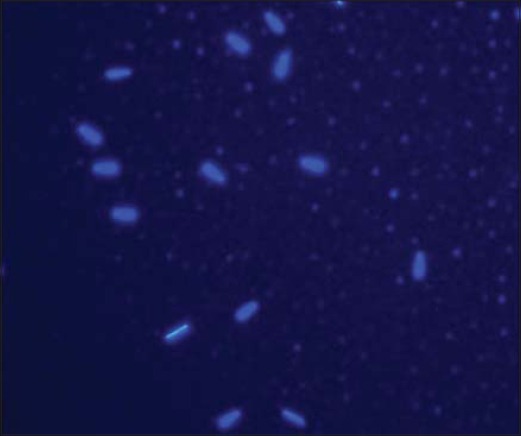
Sperm nucleus stained with DAPI (400x).

**Figure-3:**
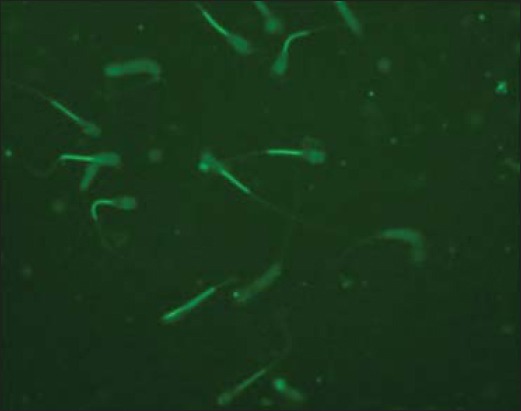
Sperm mitochondria stained with TMRM, FITC filter (400x).

Mitochondrial membrane potential was assessed using fluorescent dye tetramethylrhodamine, methyl ester (TMRM, Life Technologies; Cat# T-668). Stock solution (10 mM) was prepared in DMSO and stored at −20°C until use. A working solution of 50 μM was prepared and stored at −20°C. Semen samples (pre-freeze and post-thaw; 250 μl) were taken into microcentrifuge tubes, and 1 ml of phosphate-buffered saline (PBS) was added to them. The samples were given 2 washings with PBS by centrifuging at 1000 RPM for 5 min at 37°C. Then, 5 μl of working TMRM solution was added to each sample and incubated at 37°C for 90 min. After incubation, washing was done with 1 ml of PBS at 1000 RPM for 5 min at 37°C to remove all the unbound dye. The pellet was mixed well with 500 μl of PBS. On a micro slide, 10 μl of washed sample and 8 μl of ProLong Gold Antifade Mountant with DAPI (Life Technologies, Cat# P36941) were taken and covered with coverslip. The slide was kept at 4°C after wrapping it in aluminum foil for 10 min. The slide was examined under an upright fluorescent microscope (Nikon) with DAPI filter (420-480 nm) as shown in Figure-1, FITC filter (510-580 nm) as shown in Figure-2, and TRITC filter (530-580 nm) as shown in Figure-3. Around 100 sperms were observed for high or low fluorescence in midpiece region as an indicator of mitochondrial membrane activity.

### Evaluation of sperm phospholipase activity in Bcl-2 supplemented pre-freeze and post-thaw semen

**Figure-4:**
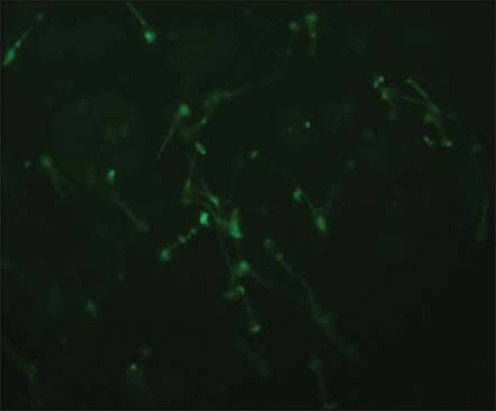
Sperm plasma membrane stained with Bodipy C11, FITC filter (400x).

Sperm phospholipid membrane was studied using BODIPY C11 fluorescent dye (4,4-difluoro-5,7-dimethyl-4-bora-3a,4a-diaza-s-indacene-3-undecanoic acid (BODIPY C11 FL, Life Technologies, Cat# D 3862). Stock solution (100 mM) was prepared in DMSO and stored at −20°C. A working solution of 20 μM was prepared and stored at −20°C. Semen samples (pre-freeze and post-thaw; 250 μl) were taken into microcentrifuge tubes, and 1 ml of PBS was added to them. The samples were given 2 washings with PBS by centrifuging at 1000 RPM for 5 min at 37°C. Then, 30 μl of working BODIPY solution was added to each sample and incubated for 45 min at 37°C. After incubation, washing was done with 1ml of PBS at 1000 RPM for 5 min at 37°C to remove all the unbound dye. The pellet was mixed well with 500 μl of PBS. On a micro slide, 10 μl of sample and 8 μl of ProLong Gold Antifade Mountant with DAPI (Life Technologies, Cat# P36941) were taken and covered with coverslip. The slides were kept at 4°C after wrapping it in aluminum foil for 10 min. Glass slides were examined under an upright fluorescent microscope (Nikon) with DAPI filter (420-480 nm) and FITC filter (510-580 nm) as shown in Figure-4 and TRITC filter (530-580 nm). Around 100 sperms in different fields were observed, and normal sperm without fluorescence was calculated and taken as % sperm with low PLA1 and PLA2 activity.

### Statistical analysis

The arcsine transformation of percent data was carried out. The data were analyzed with one-way analysis of variance and Games-Howell *post-hoc* test using IBM SPSS Version 20. The data are presented as means and standard errors for all variables. p<0.05 was considered as significant.

## Results

In our study, Tris extender was supplemented with Bcl-2 protein in the final concentration at 5, 10, and 15 µM and evaluated the pre-freeze and post-thaw semen samples in terms of percent individual motility, viability, HOST reactive sperms, mitochondrial membrane activity, and sperm PLA activity status. Data obtained was analyzed and presented in Table-1.

**Table-1:** Effects of supplementation of Bcl-2 protein at various concentrations on semen quality at pre-freeze and post-thaw stage.

Parameters (%)	Pre-freeze				Post-thaw			

	Control	5 µM	10 µM	15 µM	Control	5 µM	10 µM	15 µM
Motility	85±8.11	89.0±8.31	84.0±7.46	85.0±8.36	45.8±7.35	40.29±7.53	33.41±5.62	40.23±6.22
Live	89.19±5.56	92.2±5.89	80.5±4.22	82.12±7.60	51.6±5.77^a^	74.29±4.23^b^	49.57±8.72	53.32±7.57
HOST	84.76±8.83	71.2±4.65	73.5±6.56	67.2±4.76	50.7±6.98^a^	63.1±6.73^b^	42.43±5.66	37.76±5.88
Active mitochondria	80.11±5.77	83.6±4.67	85.13±6.34	91.22±3.94	67.1±2.44	79.3±4.67	72.4±2.33	78.5±3.68
Low PLA activity	85.23±5.43	75.45±6.37	81.35±4.92	85.61±5.92	60.23±4.45^a^	73.42±5.79^b^	75.51±6.22^b^	74.78±5.89^b^

Values marked with different superscripts differ significantly (p<0.05) in the row. HOST: Hypo-osmotic swelling test, PLA: Phospholipase A

### Effects of various doses of Bcl-2 protein supplementation at pre-freeze stage

In pre-freeze semen, % individual motility, viability, HOST reactive sperm, active mitochondria and sperms with low PLA activity were similar (p>0.05) between control and various supplementation groups.

### Effects of various doses of Bcl-2 protein supplementation at post-thaw stage

The % individual motility and % active mitochondria were also similar in post-thaw semen samples. The % viable (74.29±4.23 vs. 51.6±5.77) and % HOST reactive sperms (63.1±6.73 vs. 50.7±6.98) were significantly (p<0.05) higher in Bcl-2 supplemented samples (5 µM) as compared to control. The % sperms with low PLA activity (non-apoptotic) were significantly (p<0.05) higher in all supplementation doses of Bcl-2 protein, i.e., at 5 µM (73.42±5.79), 10 µM (75.51±6.22), and 15 µM (74.78±5.89) as compared to control (60.23±4.45).

## Discussion

This is the first report to minimize the apoptosis-like changes developed during semen cryopreservation by supplementing extender with Bcl-2 protein. In our study, Bcl-2 protein supplementation did not show any effect on individual motility and mitochondrial activity. However, Bcl-2 supplementation at 5 µM improved viability, membrane integrity, and minimized the PLA activity. A similar study has not been conducted to compare the results of this study. Cryopreservation affects sperm motility, vitality, DNA integrity, and increases in intracellular Ca^2+^ concentration which leads to the release of pro-apoptotic factors in the cytoplasm. The anti-apoptotic effects of Bcl-2 are well established in human sperm [16]. Bcl-2 inhibits caspase activity either by preventing the release of cytochrome C from the mitochondria and/or by binding to the apoptosis-activating factor-1 [17]. The primary role of the anti-apoptotic protein BCL-2 is to prevent the action of pro-apoptotic proteins responsible for pore formation in the mitochondria [18]. Bcl-2 and Bax ratio in sperm determines the fate of sperm where Bcl-2 inhibits and Bax promotes the apoptosis [6,7]. The apoptosis-promoting factor Bax has been detected in bovine spermatozoa, whereas anti-apoptotic factor Bcl-2 has not been detected [19]. The dynamic balance that occurs between anti-apoptotic members such as Bcl-2 and pro-apoptotic members helps determine whether the cell initiates apoptosis [20,21]. So, exogenous supplementation of Bcl-2 might have provided protective effects to sperm against apoptosis during cryopreservation. Bcl-2 protein inhibits mitochondrial and membranous pathway of apoptosis by preventing Ca^+^ influx and phospholipase dependent externalization of phosphatidylserine [22]. In normal sperm, phosphatidylserine is sequestered in the inner layer of the membrane by various translocases, which maintain asymmetry of sperm plasma membrane. Further, it has also been observed that in boar sperm with higher extra or intracellular phospholipase activity by the combined action of lipid peroxidation and Ca^2+^ on membrane phospholipids ultimately alters its structure and initiates membrane degradation [23]. Membrane lysis and cell death could result from excessive phospholipase enzyme activity [24] which ultimately reduces the semen quality.

In this study, two fluorescent staining Probes such as TMRM and BODIPY C11 FL were used. TMRM is a cell-permeant, red-orange fluorescent dye, bearing a delocalized positive charge, which readily enters the negatively charged mitochondria, where it accumulates in an inner membrane potential-dependent manner [25]. TMRM distributes itself within polarized mitochondria in a Nernstian manner. Membrane potential driven accumulation of TMRM within the inner membrane of healthy functioning mitochondria results in an increase in TMRM associated orange fluorescence. In case where the mitochondrial membrane potential collapses in apoptotic or metabolically stressed cells, TMRM dye disperses throughout the cell cytoplasm at a concentration that yields minimal fluorescence on excitation in the optimal wavelength region.

Molecular Probe BODIPY C11 FL substrate has been specifically designed to provide a continuous fluorescence response to PLA. The BODIPY C11 FL probe is incorporated in sperm membranes. The proximity of BODIPY C11 FL fluorophores on adjacent phospholipid acyl chains results in self-quenching of fluorescence, which is alleviated by PLA1 or PLA2 mediated release of a BODIPY C-11 FL-labeled fatty acid (BODIPY FL C11). Spermatozoa with deteriorated membrane and externalized phosphatidylserine are characterized by an increased lyso-phosphatidylcholine content that is likely generated by phospholipases [26]. Externalization of phosphatidylserine from the inner leaflet to the outer leaflet of membrane is considered as a sign of early apoptosis [27]. Using this probe, we assessed the apoptotic sperm with fluorescence. Sperm without fluorescence indicated low activity of phospholipase.

## Conclusion

On the basis of this study, it could be concluded that Bcl-2 protein supplementation exerts its protective effect on spermatozoa against apoptosis-like changes developed during cryopreservation. Bcl-2 protein supplementation in the extender at 5 µM improved post-thaw semen quality in terms of percent viability, HOST reactive sperms, and sperms with low PLA activity (non-apoptotic sperms).

## Authors’ Contributions

The entire work was carried out by JD, for his Masters degree. Planning and execution of work were under the supervision of AK. Data analysis was carried out by MH. The mitochondrial membrane activity was assessed under the supervision of DD and NS. The manuscript was written by JD and edited by AK. All authors read and approved the final manuscript.
